# Same Sex Marriage and the Perceived Assault on Opposite Sex Marriage

**DOI:** 10.1371/journal.pone.0065730

**Published:** 2013-06-11

**Authors:** Alexis Dinno, Chelsea Whitney

**Affiliations:** School of Community Health, Portland State University, Portland, Oregon, United States of America; University of Zaragoza, Spain

## Abstract

**Background:**

Marriage benefits both individuals and societies, and is a fundamental determinant of health. Until recently same sex couples have been excluded from legally recognized marriage in the United States. Recent debate around legalization of same sex marriage has highlighted for anti-same sex marriage advocates and policy makers a concern that allowing same sex couples to marry will lead to a decrease in opposite sex marriages. Our objective is to model state trends in opposite sex marriage rates by implementation of same sex marriages and other same sex unions.

**Methods and Findings:**

Marriage data were obtained for all fifty states plus the District of Columbia from 1989 through 2009. As these marriage rates are non-stationary, a generalized error correction model was used to estimate long run and short run effects of same sex marriages and strong and weak same sex unions on rates of opposite sex marriage. We found that there were no significant long-run or short run effects of same sex marriages or of strong or weak same sex unions on rates of opposite sex marriage.

**Conclusion:**

A deleterious effect on rates of opposite sex marriage has been argued to be a motivating factor for both the withholding and the elimination of existing rights of same sex couples to marry by policy makers–including presiding justices of current litigation over the rights of same sex couples to legally marry. Such claims do not appear credible in the face of the existing evidence, and we conclude that rates of opposite sex marriages are not affected by legalization of same sex civil unions or same sex marriages.

## Introduction

Marriage has many values to individuals and societies. The codification of marriage into U.S. Federal law alone provides over a thousand conditions in which married couples are treated differently than non-married couples. While some disadvantages may result to married couples relative to unmarried couples in these laws–as when there are married couple penalty provisions in the tax code–most of these laws provide substantive benefits to married couples relative to unmarried couples [1]. Marriage is well understood as a basic determinant of the health of adults [2] and their children [3], [4]. Married individuals are less likely than non-married individuals to report their health as fair or poor, less likely to suffer from physical ailments or report poor psychological health, and across the lifespan report fewer health ailments [5]. Marriage is associated with greater life satisfaction and improved mental health [6], [7].

Until recently same sex couples in the United States have been excluded from legally recognized marriage. The current national policy debate over same sex marriage intensified in 1993, when in the Hawaii’s Supreme Court ruled in Baehr v. Miike “that under that states constitution, a marriage statute which restricts the status and benefits of marriage to male-female couples discriminates on the basis of sex.” [8] In 1996 the federal Defense of Marriage Act (DOMA) restricted marriage to a legal union between one man and one woman, and, responding to concerns that some states would at some point be required to recognize same sex marriages from other states, gave states the power to restrict marriage to opposite sex couples and to not recognize same sex marriages from other states. Thirty states have passed state DOMAs and statute restrictions on marriage [9]. In most states, same sex couples are still excluded from marriage and all same sex couples are excluded from the federal benefits of marriage.

Massachusetts became the first state to allow same sex marriages on May 17, 2004 following the ruling in Goodridge v. Department of Public Health (440 Mass. 309 Mass: Supreme Judicial Court, 2003). Subsequently, Connecticut (November 12, 2008), Iowa (April 27, 2009), New Hampshire (January 1, 2010), New York (July 24, 2011), Vermont (September 1, 2009), Washington (December 6, 2012), Maine (December 29, 2012), Maryland (January 1, 2013) and the District of Columbia (December 18, 2009) have joined Massachusetts in legalizing same sex marriages (see Table S1 in File S1). California’s Supreme Court ruled in 2008 that prohibiting same sex couples from marrying was unconstitutional (In re MARRIAGE CASES, 2008, 43 Cal.4th 757). Same sex marriages were allowed in California between June 17th, 2008 and November 4th, 2008 during which time approximately 18,000 couples were married [2]. In November of 2008, CA voters passed Proposition 8 [10] defining marriage as one man and one woman. While the federal lawsuit challenging California’s Proposition 8 is working its way through the appeals process (See: Perry v. Brown, No. 10–16696, 9th Cir. Feb 7, 2012), the 18,000 CA same sex marriage licenses issued in 2008 remain valid (Strauss v. Horton, 2009, 46 Cal.4th 364).

In 2000, Vermont became the first state to allow civil unions for same sex couples following a supreme court ruling that marriage benefits could not be restricted to opposite sex couples (Baker v. Vermont, 744 A. 2d 864 Vermont: Supreme Court, 1999). Following Vermont, eleven states, including California, Connecticut, Delaware, Hawaii, Illinois, Nevada, New Hampshire, New Jersey, Oregon, Rhode Island, and Washington as well as the District of Columbia enacted legislation recognizing same sex ‘domestic partnerships’ or ‘civil unions’ which do or did extend most or all of the state-level benefits of marriage, explicitly reserving the legal designation of marriage to opposite sex couples (see Table S1 in File S1). Several states, including Colorado, Maine, Maryland, Wisconsin, and previous to stronger same sex union laws, in California, the District of Columbia, New Jersey and Washington enacted legislation recognizing same sex ‘domestic partnerships’ or ‘designated beneficiaries,’ which have provided a limited subset of state-level benefits of marriage to registered couples (see Table S1 in File S1).

### Is Same Sex Marriage a Detriment to Opposite Sex Marriage?

Opponents to legalization of same sex marriage have positioned it as an “assault” [11] seeking to “weaken,” [12] “destroy” [13]–[16] and “undermine” [17], [18] opposite sex marriage. Anti-same sex marriage lawmakers, advocates, and journalists have raised concerns over the social effects of legalizing same sex marriage. One such use of language has positioned same sex marriage as literally harmful to opposite sex marriage: in a recent ruling of the United States Court of Appeals for the Ninth Circuit in Perry the proponents argue “if the definition of marriage between a man and a woman is changed, it would fundamentally redefine the term from its original and historical procreative purpose. This shift in purpose would weaken society’s perception of the importance of entering into marriage to have children, which would increase the likelihood that couples would choose to cohabitate rather than get married” (Perry v. Brown, No. 10–16696, 111-112–9th Cir. Feb 7, 2012). David Blankenhorn, an expert witness for the defendants in Perry testified under oath “that allowing same-sex marriage would undermine respect for the unique status of traditional marriage, and this could lead to further deinstitutionalization, including an increase in out-of- wedlock births, divorce, etc” [19]. The argument that same sex marriage literally destroys opposite sex marriages translates directly to the question of what has happened to rates of opposite sex marriage in states that allow same sex marriage as compared to other states which do not? A similar question has been posed in the academic arena with respect to opposite sex marriage rates in Denmark, Norway, Sweden, Iceland, and the Netherlands, and no significant change in opposite sex marriage and divorce rates following enactment of same sex marriage laws was found [20]. The academic literature quantitatively assessing the effect of same sex marriage laws on rates of opposite sex marriage in the U.S. is tiny, with, we believe, just one study that analyzed a static model of marriage rates from three years (1990, 2000, and 2004) and found a significant positive association between “gay marriage, or full legal recognition like civil unions” and state marriage rates [21].

Despite the argument that legalizing same sex marriage will decrease the rates of opposite sex marriage, some opposite sex couples in the U.S. are currently boycotting marriage until it is available to all [22], [23]. Heterosexual and bisexual individuals and opposite sex couples across the country have pledged to boycott marriage until it is available to all by joining the National Marriage Boycott, started after the passage of Proposition 8 [24]. The movement has been joined by churches as well who have stopped signing marriage licenses in support of marriage equality [25], [26]. That some opposite sex couples will not marry unless same sex marriages are lawful suggests, contrary to the prognostications of some opponents of same sex marriage, that a probable increase in marriage rates over time will follow the legalization of same sex marriage. The fact that some opposite sex couples are postponing marriage until it is legal also for same sex couples implies that there may also be a limited period of increase in opposite sex marriages following enactment of same sex marriage laws. A helpful anonymous reviewer of this article conjectures that same sex marriage laws could be expected to have two kinds of effects on rates of opposite sex marriage. Because by legitimizing same sex relationships, same sex marriage laws could help reduce the number of homosexuals living closeted lives and entering into unhappy opposite sex marriages, such laws might both contribute to decreased numbers of new opposite sex marriages, but also reduce the number of opposite sex marriages likely to end in divorce because the marriage was undertaken to keep up heterosexual appearance by a homosexual participant. Therefore caution must be taken about conflating causes of state-level rates of opposite sex marriage with causes of individual-level or couple-level participation in opposite sex marriage.

We aim to test the claims that rates of opposite sex marriage will change as a result of same sex marriage or strong or weak same sex union laws. Our primary formal hypothesis is twofold: (1) that there is in the short or long-term a decreasing trend in rates of opposite sex marriage following implementation of same sex marriage laws, and (2) that states enacting same sex marriage laws experience an increase in opposite sex marriages in the short-term following implementation. These primary hypotheses are accompanied by four parallel secondary hypotheses for comparable short-term and long-term effects following implementation of strong same sex union laws providing most or all of the benefits of marriage excepting the term marriage, and for weak same sex union laws providing a small subset of the benefits of marriage.

## Materials and Methods

We model marriage rates in the thirteen states plus the District of Columbia where same sex marriage or strong or weak same sex union laws were implemented before 2009 relative to rates in the remaining states..

### Variables and Data

Marriages by state and year from 1988 to 2009 were obtained from National Center for Health Statistics (NCHS) marriage publications [27]–[41], excepting Louisiana in 2006 when NCHS data were unavailable. We used the Louisiana Department of Health and Hospitals marriage rate figure for 2006 because NCHS marriage figures from 2005 and 2007 are identical to the Louisiana Department of Health and Hospitals figures for those same years [42]. Mid-year (July, 1) estimates of the U.S. population 18 years and older by state were obtained from the U.S. Census Bureau Population Estimates historical data by state (http://www.census.gov/popest/data/historical/). The adult population in each state was used as this represented those ‘at risk’ of marriage for purposes of analytic precision (and not intended as a substantive redefinition marriage rate). The total number of marriages in each study state were adjusted downward by the corresponding number of same sex marriages [43]–[46] appropriate to each year from enactment to 2009. Because California did not track same sex marriages in 2008, we used the widely-reported figure of 18,000 same sex marriages in California during 2008 [2]. Marriage rates were calculated as all control states marriages minus the total number of reported same sex marriages (i.e. zero in most states and years), divided by the in-state adult population at mid-year. The sample size was 1071.

Data for state same sex marriage, and strong and weak same sex union laws were taken from public legislative and court records (see Data S1). In each year, same sex marriage and union laws were separately encoded in each state with a proportion representing how much of that year the law was in effect. For example, Massachusetts implemented same sex marriage on May 17, 2004, so during the first year following enactment the same sex marriage variable for this state had the value 0.623 in 2004, the value 1.0 in all subsequent years, and the value 0.0 in all previous years. A multiplicative interaction term for same sex marriages and strong same sex unions to capture those occasions when both laws were in force simultaneously.

### Missing Data

Marriage data were missing for California in 1991 and for Oklahoma for 2000–2004. The portion of missing marriage data was 0.0045. We accounted for increased uncertainty in our estimates due to data missingness using bootstrap estimation maximization multiple imputation methods developed for missing time series data with the amelie package version 1.5–5 for R. version 2.14. [47] Reported are the results of identical analyses on ten imputed data sets combined [48] to reflect increased uncertainty due to data missingness. See File S1 for further details.

### Non-stationarity of Marriage Rates

A first-lag random intercept model (1) provided an estimate of 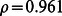 (95% CI:0.953, 0.970), suggesting that marriage rates during the study period were strongly autoregressive and near-integrated (i.e. non-stationary) processes [49], [50]. Application of Hadri’s test for unit root in panel data allowing for cross-sectional dependence and subtracting cross-sectional means [51] confirmed that marriage rates in some states were neither trend stationary (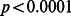) nor level stationary (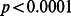). The Im-Pesaran-Shin test for unit root with a single lag and subtracting cross-sectional means [52] failed to reject the null hypothesis that all states contain unit roots both with time trend (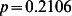) and without (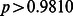).
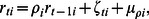
(1)where:


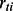
 is the marriage rate at time 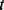 in state 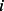,


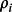
 measures autocorrelation and is permitted to vary for each state,


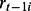
 is the first lag of the marriage rate in each state,


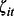
 measures all disturbances to 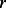 in each time 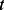 (assumed distributed normal), and


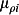
 measures state-level variation in 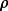 (assumed distributed normal).

### Data Analysis

We modeled state-level differences in opposite sex marriage rates by differences in their enactment of same sex marriage laws and strong and weak same sex union laws. Because marriage rates are near-integrated, stationary models of change in marriage rates cannot provide reliable estimates [53]. Instead, change in marriage rates in year 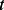 and state 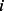 was fit using a single-equation generalized error correction model (GECM) [49], [50] (equation 2), permitting inference about the short term and long term effects on opposite sex marriage rates of same sex marriage and union laws. The GECM is an appropriate model both because GECMs are appropriate for modeling near-integrated outcome variables irrespective of a co-integration between outcome and predictor variables [50], [54], and because we infer that same sex marriage, and strong and weak same sex unions all have level unit root (same sex marriage and strong same sex unions have trend unit root, although in some states weak same sex unions may be stationary) from both Hadri’s test allowing for cross-sectional dependence and subtracting cross-sectional means and the Im-Pesaran-Shin test with a single lag and subtracting cross-sectional means. The interaction term, 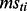, is stationary (see discussion of the homogeneity of the error correction process in the discussion).

The random intercept term, 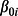, was permitted to vary by state, both to reflect the fact that states have different average changes in marriage rates at equilibrium (i.e. it would be unreasonable to fit the model by assuming, for example, that Hawaii and Mississippi experience similar changes in marriage rates), and in order to produce more accurate standard error estimates of the fixed effect parameters.
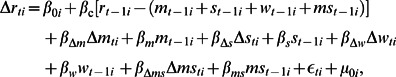
(2)where:


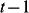
 in the subscript indicates the first lag for a variable in year t;


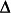
 is the one-year change function for a variable (e.g. 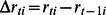);


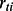
 is the marriage rate in year 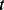 in the 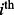 state;


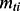
 is the proportion of year 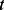 that same sex marriage laws were in force in the 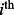 state;


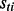
 is the proportion of year 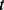 that strong same sex union laws were in force in the 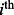 state;


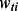
 is the proportion of year 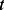 that weak same sex union laws were in force in the 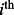 state;


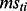
 is the multiplicative interaction of 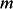 and 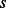 in year 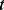 in the 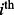 state;


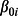
 is the model constant for the 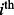 state;


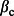
 is the ‘correction rate’ at which marriage rates return to equilibrium after a perturbation;


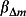
 is the ‘short run instantaneous effect’ of same sex marriage law implementation in the absence of concurrent strong same unions (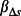, 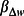, and 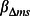 are the ‘short run instantaneous effects’ of the respective covariates);


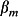
 is the ‘lagged effect’ of same sex marriage law implementation in the absence of concurrent strong same unions (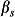, 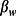, and 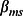 are the ‘lagged effects’ of the respective covariates);


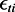
 is the residual at time 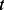 in the 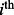 study state;


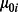
 is the model constant term for the 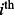 study state, and where 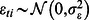, and 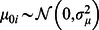.

The parameters in (2) provide different possible interpretations of our hypotheses in the form of short and long term effects of same sex marriage and strong and weak same sex union laws on opposite sex marriage rates. Short run instantaneous effects are given by 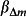, 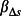, and 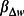 and, for same sex marriages concurrent with strong same sex unions, by (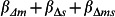). Short run lagged effects (for example, for marriage in the absence of concurrent strong same sex union laws) are given by 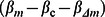, and (for same sex marriages concurrent with strong same sex unions) by 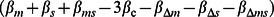. Finally, long run effects (for example, for marriage in the absence of concurrent strong same sex union laws) are given by 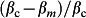, and (for same sex marriages concurrent with strong same sex unions) by 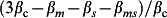. We estimated the model in equation (2) for all fifty states plus the District of Columbia in order to evaluate the short and long term effects of same sex marriage and union laws against opposite sex marriage rates in control states using the xtmixed command in Stata version 11.2. Estimates and standard errors for long run effects, lagged short run effects and the instantaneous short run combined effect of same sex marriages contemporaneous with strong same sex unions were calculated using the delta method using the nlcom command in Stata.

## Results

**Figure 1:**
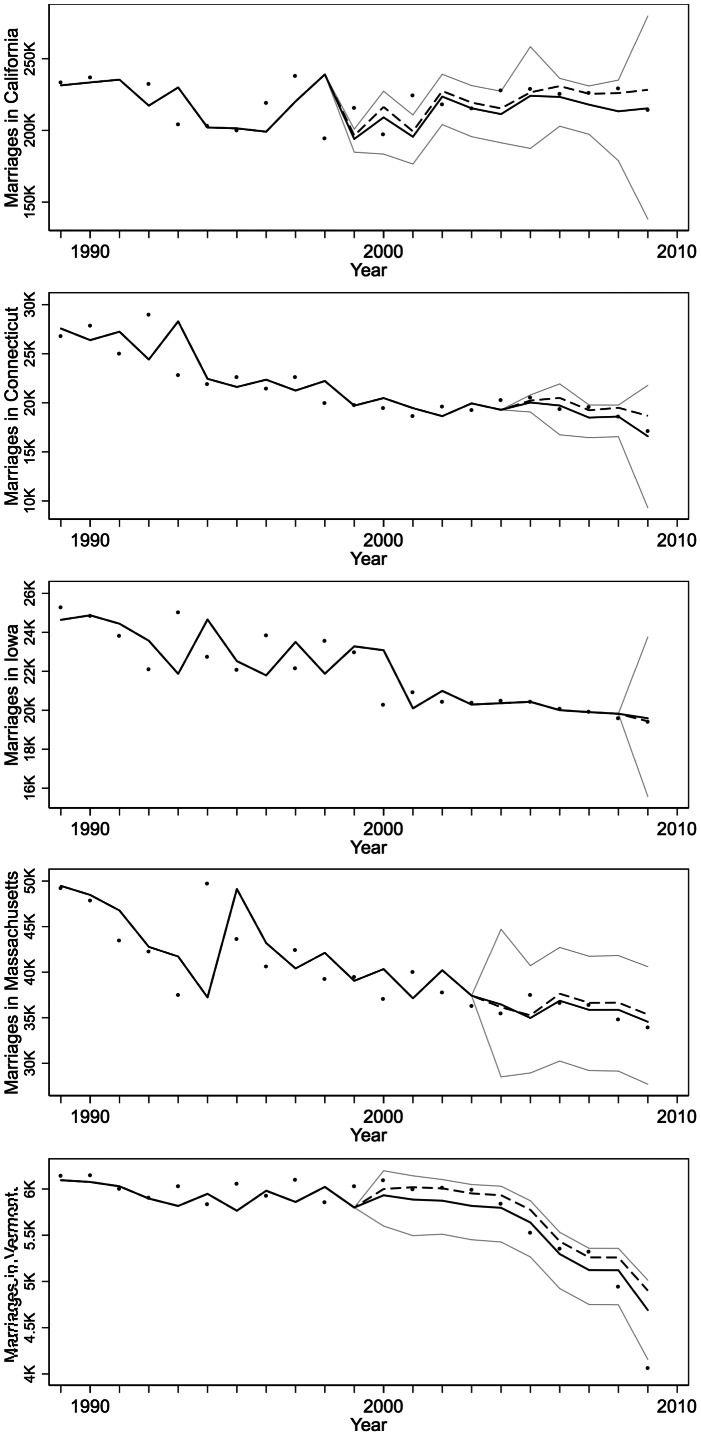
Projected differences in annual opposite sex marriages in states enacting same sex marriage laws. Solid black lines represent our modeled marriages in each year and state, and dashed black lines project opposite sex marriages if same sex marriage laws had not been enacted in each state and year. Observed numbers of marriages are plotted as dots–note that the model follows very closely on the previous year’s observed number of marriages. The 95% confidence intervals of the difference in predicted opposite sex marriages with and without same sex marriage laws in effect are centered on the average of those two predictions. California licensed 18000 same sex marriages in 2008. Connecticut enacted a same sex marriage law in 2008. Iowa enacted a same sex marriage law in 2009. Massachusetts enacted a same sex marriage law in 2004. Vermont enacted a same sex marriage law in 2009.

All short term and long term effects of same sex marriages and strong and weak same sex unions were close to zero and statistically undifferentiable from the null hypothesis of no effect on rates of opposite sex marriage with %95 confidence intervals uniformly spanning zero (Table 1). This finding holds even for very large values of 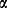. Of course absence of evidence, is not the same thing as evidence of absence [55]. Therefore we also performed equivalence hypothesis tests on each of the dynamic effects reported in Table 1 by posing as null hypotheses *differences* between the reported effects and zero within a given tolerance, 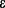, deciding whether to reject them in favor of alternative hypotheses of effects within the range 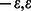 by using uniformly most powerful tests of equivalence [56]. We employed and report results for liberal (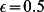), strict (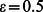) and very strict (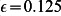) tolerance values (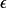 is measured in units of 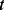, see, for example, page 16 of [56]). The results of the equivalence tests (Table 2) were unambiguous: we rejected all null hypotheses of difference in of the dynamic effects of favor of equivalence to no effect for liberal, strict and very strict tolerances. In Table 2 we report 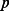-values adjusted for the False Discovery Rate (FDR) [57] only for 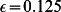, as the FDR adjustments make no difference within the precision of of the reported figures for 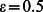 or 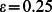. Thus, we found that adult rates of opposite sex marriage in states implementing same sex marriage laws, both with and without contemporaneous strong same sex union laws, were equivalent to rates in states with no such laws, and we find that any differences appear to due to chance alone, as reflected in very wide confidence intervals around the predicted differences in states implementing same sex marriage laws (Figure 1). Figure S1 in File S1 shows graphs for all states with any same sex marriage or same sex union laws. The raw model parameter estimates and standard errors from (2) are presented in Table S2 in File S1.

**Table 1:** Effects of same sex marriage and union laws on opposite sex marriage rates (N = 1071).

	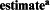	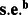	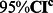	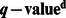
*Instantaneous short run effects of*				
same sex marriage w/o strong unions	0.0001	0.0013	−0.0025, 0.0027	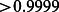
same sex marriage & strong unions	-0.0007	0.0014	−0.0035, 0.0021	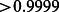
strong same sex unions w/o marriage	-0.0003	0.0007	−0.0016, 0.0010	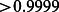
weak same sex unions	-0.0004	0.0006	−0.0016, 0.0008	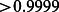
*Lagged short run effects of*				
same sex marriage w/o strong unions	-0.0003	0.0015	−0.0031, 0.0026	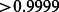
same sex marriage & strong unions	-0.0004	0.0031	−0.0064, 0.0056	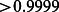
strong same sex unions w/o marriage	0.0000	0.0007	−0.0014, 0.0014	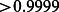
weak same sex unions	0.0002	0.0007	−0.0011, 0.0015	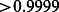
*Long run run effects of*				
same sex marriage w/o strong unions	-0.0037	0.0152	−0.0335, 0.0261	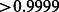
same sex marriage & strong unions	-0.0279	0.0754	−0.1756, 0.1199	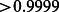
strong same sex unions w/o marriage	-0.0067	0.0075	−0.0215, 0.0081	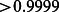
weak same sex unions	-0.0036	0.0083	−0.0199, 0.0127	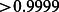

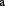
The arithmetic mean of the estimates from all ten imputed data sets.

Combined standard errors account for both within- and between-imputation estimate variance.

95% confidence intervals are given by the estimate 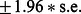.

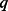
-values are 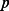-values adjusted upward to account for twelve multiple comparisons; compare to 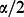.

**Table 2:** Equivalence tests for dynamic effects on opposite sex marriage rates (N = 1071).

	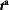	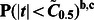	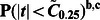	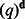 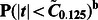
*Instantaneous short run effects of*				
same sex marriage w/o strong unions	0.0741	0.0000	0.0000	0.0078 (0.047)
same sex marriage & strong unions	−0.5095	0.0000	0.0000	0.0191 (0.023)
strong same sex unions w/o marriage	−0.4456	0.0000	0.0000	0.0176 (0.023)
weak same sex unions	−0.5782	0.0000	0.0000	0.0208 (0.023)
*Lagged short run effects of*				
same sex marriage w/o strong unions	−0.1730	0.0000	0.0000	0.0108 (0.032)
same sex marriage & strong unions	−0.1435	0.0000	0.0000	0.0099 (0.040)
strong same sex unions w/o marriage	0.0181	0.0000	0.0000	0.0051 (0.061)
weak same sex unions	0.3044	0.0000	0.0000	0.0141 (0.028)
*Long run run effects of*				
same sex marriage w/o strong unions	−0.2426	0.0000	0.0000	0.0126 (0.030)
same sex marriage & strong unions	−0.3700	0.0000	0.0000	0.0270 (0.027)
strong same sex unions w/o marriage	−0.8857	0.0000	0.0000	0.0286 (0.029)
weak same sex unions	−0.4364	0.0000	0.0000	0.0260 (0.026)

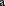
The quotient of the Table 1 estimates and their standard errors.

The critical value 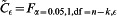 where 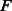 is a quantile function of the noncentral 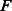-distribution, the degrees of freedom are 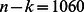 from equation 2, and 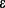 is the noncentrality parameter of 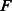, and the 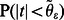 is the cumulative density of 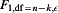 at 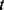 [56]. Because under the null hypothesis of difference, one of the two single-tails of the tests must be rejected, these 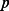-values should be compared to 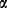 rather than to 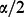 for the common interpretation of false rejection under null hypotheses of difference [56], [60].

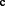
The 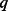-values for 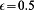 and 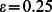 are not explicitly reported because the figures remain just as the 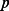-values within the precision of this table.

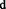
 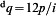, where 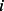 is the position of ordered 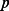-values from smallest to largest. When stepping down from largest to smallest 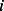, all hypotheses are rejected including and subsequent to the first with 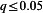 to control the FDR for twelve multiple comparisons.

Across analyses of all ten imputed data sets, Hadri’s test for unit root for panel data allowing for cross-sectional dependence and subtracting cross-sectional means [51] failed to reject both the null hypothesis that the error terms from all states were trend stationary (mean 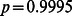) and the null hypothesis that the error terms from all states were level stationary (mean 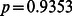): we conclude that our model was appropriate to test our hypotheses.

Models models with additional lags including up through the fourth lags of marriage rates gave substantively similar results with no difference in inferences from Tables 1 and 2.

## Discussion

We found that state rates of opposite sex marriage in the U.S. from 1989–2009 do not significantly differ when same sex marriage and union laws are in force compared to when they are not in force, contrary both to concerns raised by opponents of same sex marriage and same sex civil unions, and to the positive association reported by Langbein and Yost [21]. We found no evidence of an increase in state-level opposite sex marriage rates corresponding to a first year effect of same sex marriage, contradicting the marriage equality hypothesis. Indeed, per our equivalence tests, we found evidence of an absence of any effects. Our analysis allows inference into changes in opposite sex marriage rates by year and state, but we cannot readily translate this inference into relationships between opposite sex couple-level marriage decisions and state-level policies without committing the ecological fallacy [58], [59]. Given the nuances we raised in the background section regarding individuals’ and couples’ motivations for choosing to marry a partner of the opposite sex or not, it is clear that only further research including both individual-level and state-level data will illuminate the effects of state marriage laws on individuals’ and couples’ marriage choices. Such a study could also examine the psychological effects of anticipated changes to marriage law on marriage behavior.

The question of whether states ought to legally provide same sex couples with the legal status of marriage, or a related, though less regarded and less beneficial status of same sex union cannot be answered solely in terms of the effect on opposite sex marriages. However, a deleterious effect on rates of state rates of opposite sex marriage has been argued to be a motivating factor for both the withholding and the elimination of existing rights of same sex couples to marry by policy makers–including presiding justices of current litigation over same sex couples rights to legally marry. Such claims do not appear credible in the face of the existing evidence.

We began by framing marriage as a social determinant of health. Marriage is an important social resource for the health of both opposite sex and same sex couples, and their children. If rates of opposite sex marriage are threatened by same sex marriage, then part of the societal measure of that threat is the limiting of a basic resource for the health of opposite sex couple-based families (through, for example, pension benefits, hospital visitation rights, immigration rights, child support, medical benefits due married partners, affordable housing benefits, etc.) who remain unmarried. This view is not supported by our findings. Conversely, if rates of opposite sex marriage are not threatened by same sex marriage, then the denial of marriage rights to same sex couples is a denial of a basic resource for the health of same sex couple-based families. This view is supported by our findings.

### Limitations

More states currently have same sex marriage and union laws in force than during our study period. Including such states would provide greater precision in our estimates, and potentially permitting an positive assessment of both the marriage equality hypothesis and the threat to opposite sex marriage hypothesis. Unfortunately there is a trend away from reporting the number of marriages by state at the national level, and in many states, making later data more difficult to obtain.

Our analysis assumes no state-level confounding factors are biasing the estimates of the effects of same sex marriage and union laws. This is appropriate in that our hypotheses were directly informed by conjectures and assertions within a recent and ongoing nation-wide discussion on the legitimacy of providing or denying same sex couples the right to legally recognized marriage, and this discourse has not generally been characterized by conjecture about confounding effects. For example, presiding justices making the argument that same sex marriage could discourage opposite sex marriage have not suggested that this effect varies depending on economic conditions, or on demographic makeup within a state. However, further research in the subject may produce insights in examining such possibilities both at the state and individual level.

Our model assumes that the effects of same sex marriage and union laws on change in rates of opposite sex marriage do not differ by state. If this assumption poorly reflects the reality (e.g. same sex marriages increase rates of opposite sex marriage in some states, but decrease rates of opposite sex marriage in other states), we may be blind to nuances of the cultural force of same sex marriages and unions. Unfortunately, the size of the current data set, in particular, the limited number of states and years implementing same sex marriage or union laws, provides poor power to discriminate random effects at the state level. Relatedly, differences in same sex marriage or same sex union laws in neighboring states might produce cross-border marriage effects which our data and study design cannot readily address. This is a complex issue, for many reasons: some states require residency for a marriage; there is likely limited legal benefit to being married in another state when it is illegal in one’s own; the role of geographic isolation (e.g. California versus Rhode Island) in limiting travel. While such ‘marriage migration’ may mismatch the numerator (marriages) from the denominator (marriageable-age population), the random intercept term 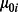 captures state-specific differences in marriage rates which are relatively constant across the study’s duration.

We also made an assumption of homogeneity of error correction rates by state, and by same sex marriage or union laws. This assumption appears reasonable for two reasons. First, the error correction process is dominated by the first lag of marriage rates, and the lagged same sex marriage and union terms cancel with it to produce near-zero estimates. Second, models accounting for only one kind of the same sex marriage, strong, or weak same sex union laws (see Tables S3–S8 in File S1) produced very similar values for 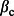 as that which we report here.

Ideally, we would have wanted to extend this analysis to divorce: inherent in the critiques against same sex marriage described above are concerns about opposite sex divorce. For example, former Arkansas Governor Mike Huckabee articulated this perspective against same sex marriage clearly “There is a quantified impact of broken families” [13]. However, many more divorce data are missing: twelve states are missing divorce data from 1990–2009–California, Indiana, and Louisiana in particular are missing most years’ data–and the overall rate of missingness is 7.93%. In addition, we encounter an analytic conundrum with divorce rates by state, which present neither uniformly stationary nor uniformly near-integrated processes, making the appropriate choice of model unclear.

### Conclusion

We conclude that there is no relationship between implementation of same sex marriage or strong or weak same sex union laws and rates of opposite sex marriage. Because the history of same sex marital rights is young in the U.S., ongoing examination of these relationships is warranted.

## Supporting Information

File S1 Supporting Information File S1 is a word processing document (in.docx format) containing Table S1: State same sex marriage and strong and weak same sex union laws; details of the imputations, including equations S1–S3; Table S2: Fixed and random effect model estimates of change in opposite sex marriage rates by state and year; Figure S1 Projected differences in annual opposite sex marriages in states enacting same sex marriage or strong or weak same sex union laws; separate generalized error correction models for same sex marriage and strong and weak same sex union laws, including equations S4–S6; Table S3: Effects of only same sex marriage laws on opposite sex marriage rates; Table S4: Fixed and random effect model estimates of change in opposite sex marriage rates by state and year for same sex marriage only; Table S5: Effects of only strong same sex union laws on opposite sex marriage rates; Table S6: Fixed and random effect model estimates of change in opposite sex marriage rates by state and year for strong same sex unions only; Table S7: Effects of only weak same sex union laws on opposite sex marriage rates; Table S8: Fixed and random effect model estimates of change in opposite sex marriage rates by state and year for weak same sex unions only; and References S1.
(DOCX)

Data S1 Supporting Information Data S1 is a spreadsheet (in.xlsx format) containing Sheet S1: Reported US marriages by state and year (annotated); Sheet S2: Reported number of US same sex marriages by state and year; and Sheet S3: Estimated US population age 18+ by state and year: US Bureau of the Census.
(XLSX)
