# Supplementary material for: Same Sex Marriage and the Perceived Assault on Opposite Sex Marriage
Source: PLoS One. 2013 Jun 11;8(6):e65730. doi: 10.1371/journal.pone.0065730 (PMC3679150; doi:10.1371/journal.pone.0065730)
Supplement: File S1 — Supporting Information File S1 is a word processing document (in.docx format) containing Table S1: State same sex marriage and strong and weak same sex union laws; details of the imputations, including equations S1–S3; Table S2: Fixed and random effect model estimates of change in opposite sex marriage rates by state and year; Figure S1 Projected differences in annual opposite sex marriages in states enacting same sex marriage or strong or weak same sex union laws; separate generalized error correction models for same sex marriage and strong and weak same sex union laws, including equations S4–S6; Table S3: Effects of only same sex marriage laws on opposite sex marriage rates; Table S4: Fixed and random effect model estimates of change in opposite sex marriage rates by state and year for same sex marriage only; Table S5: Effects of only strong same sex union laws on opposite sex marriage rates; Table S6: Fixed and random effect model estimates of change in opposite sex marriage rates by state and year for strong same sex unions only; Table S7: Effects of only weak same sex union laws on opposite sex marriage rates; Table S8: Fixed and random effect model estimates of change in opposite sex marriage rates by state and year for weak same sex unions only; and References S1. (DOCX) [file pone.0065730.s001.docx]

**Supporting Information File S1**

Table S1: State same sex marriage and strong and weak same sex union laws

| **State or District** | **Weak same sex unions** | | **Strong same sex unions** | | **Same sex marriages** | |
| --- | --- | --- | --- | --- | --- | --- |
|  | **Dates in effect** | **Law citations** | **Dates in effect** | **Law citations** | **Date in effect** | **Law citations** |
| CA | 9/22/99 | Domestic Partnership Act of 1999, A.B. 26, CA 1999-2000, (1999).; Expanded Rights in Domestic Partnerships: A.B. 26, CA 2001-2002, (2001). | 1/1/05 | California Family Code: Domestic Partner Registration, 2.5 Cal. Code § 297-297.5 | 6/16/08  11/5/08 | California Family Code: Domestic Partner Registration, 2.5 Cal. Code § 297-297.5; C.A. Const. art. I, § 7.5; *In re Marriage Cases*, 43 Cal.4th 757 (2008). |
| CO | 7/1/09 | Colorado Designated Beneficiary Agreement Act, 15 Colo. Rev. Stat. § 22 (2009) [Not counted as a ‘civil union’ in our analysis.] |  |  |  |  |
| CT |  |  | 10/1/05–10/1/10 | An act concerning civil unions, SB963, CT 2005, (2005). | 11/12/08 | Marriage, 815 Conn. Gen. Stat. § 46b-20a (2009); *Kerrigan v. Commissioner of Public Health*, 957 A. 2d 407 Conn: Supreme Court (2008) |
| DC | 3/4/02 | Health Care Benefits Expansion Act of 1992, DC Law-9-114, DC Legislative Council 1992, (1992); Non-marriage opposite sex unions in effect 3/4/2002 | 1/4/06 | Local Business Affairs: Labor, 32 D.C. Code. § 32-702 | 3/3/10 | Domestic Relations: Marriage, 46 D.C. Code. § 46-401 |
| DE |  |  | 1/1/12 | Civil Unions, 13 Del. C. § 201 |  |  |
| HI |  |  | 1/1/12 | Civil Unions, 572 Haw. Rev. Stat.§ 572B; Non-marriage opposite sex unions in effect 1/1/2012 |  |  |
| IL |  |  | 6/1/11 | Illinois Religious Freedom Protection and Civil Union Act, 750 Ill. Comp. Stat. § 75.1-75.90; Non-marriage opposite sex unions in effect 6/1/2011 |  |  |
| IA |  |  |  | Domestic Relations: Marriage, 595 Iowa Code § .1-.21; *Varnum v. Brien* 763 NW 2d 862 Iowa: Supreme Court (2009) | 4/27/ 09 |  |

Table S1: State same sex marriage and strong and weak same sex union laws, continued

| **State or District** | **Weak same sex unions** | | **Strong same sex unions** | | **Same sex marriages** | |
| --- | --- | --- | --- | --- | --- | --- |
|  | **Dates in effect** | **Law citations** | **Dates in effect** | **Law citations** | **Date in effect** | **Law citations** |
| ME | 6/30/04 | Domestic Partner Registry, 22 Me. Rev. Stat. Ann. §2710; Non-marriage opposite sex unions in effect 7/30/2004 |  |  | 12/29/12 | Voters approved gender-neutral legal definition of marriage. Sec. 1. 19-A Me. Rev. Stat. Ann. §650-A |
| MD | 7/1/08 | Maryland Health General: Domestic partners, 6 Md. Code Ann. § 6-101; Non-marriage opposite sex unions in effect 7/1/2008 |  |  | 1/1/13 | Maryland Family Law: Marriage, 2 Md. Code Ann. § 2-201  (2012), voters upheld the law, despite an attempted repeal through referendum Question 6 defeated 11/6/12. |
| MA |  |  |  |  | 5/17/04 | Domestic Relations: Marriage, 3, Mass. Gen. Laws § 207.1-207.58; *Goodridge v Dept of Public Health*440 Mass. 309 Mass: Supreme Judicial Court (2003) |
| NV |  |  | 10/1/09 | Nevada Domestic Partnership Act, 122.A Nev. Rev. Stat. §100; Non-marriage opposite sex unions in effect 10/1/2009 |  |  |
| NH |  |  | 1/1/08 | Domestic relations: Civil Unions, 43 N.H. Rev. Stat. Ann. § 457-A (repealed) | 1/1/10 | Equal Access to Marriage, 43 N.H. Rev. Stat. Ann § 457:1-a (2009) |
| NJ | 7/10/04 | Domestic Partnership Act, S2820 & A3743, NJ Legislature 2002-2003, (2003). | 2/19/07 | Marriages and Married Persons, 37 N.J. Stat. Ann. § 37:1-31 (2006) |  |  |
| NY |  |  |  |  | 7/24/11 | Domestic Relations: Parties to a marriage, 3 N.Y. Laws § 10-a |
| OR |  |  | 2/4/08 | Oregon Family Fairness Act, 11 O.R. Rev. Stat.§ 106.300-106.340 |  |  |
| RI |  |  | 7/1/11 | Civil Unions, 15 R.I. Gen. Laws. § 3.1.1-3.1.11 |  |  |
| VT |  |  | 7/1/00 | Domestic Relations: Civil Union, 15, Vt. Stat. Ann. § 23.1201-23.1207; *Baker v. Vermont* 744 A. 2d 864 Vermont: Supreme Court (1999) | 9/1/09 | Domestic Relations: Civil Marriage, 15 Vt. Stat. Ann. § 1.8 (2009) |

Table S1: State same sex marriage and strong and weak same sex union laws, continued

| **State or District** | **Weak same sex unions** | | **Strong same sex unions** | | **Same sex marriages** | |
| --- | --- | --- | --- | --- | --- | --- |
|  | **Dates in effect** | **Law citations** | **Dates in effect** |  | **Dates in effect** | **Law citations** |
| WA | 7/22/07 | Protecting individuals in domestic partnerships by granting certain rights and benefits, SB 5336, WA Legislature 2007, (2007); Expanded Rights in Domestic Partnerships: Expanding rights and responsibilities for domestic partnerships, H.B. 3104, WA Legislature 2008, (2008). | 12/3/09 | State Registered Domestic Partnerships, 26 Wash. Rev. code § 60.010-60.901 | 12/6/12 | A law recognizing same sex marriages was passed by the state legislature and signed by the governor. See Marriage Contract, 26 Wash. Rev. Code § 04.010 (2012). However, this law required approval of the voting public through Referendum 74 which occurred on November 6, 2012. |
| WI | 8/3/09 | Domestic Partnership, 770 Wis. Stat. § 770.001-770.18 |  |  |  |  |

*Details of imputation*

Five observations (0.45%) of total number of marriages (and, therefore, marriage rates) in states and years were missing (California in 1991, and Oklahoma 2000–2003). Expression S1 outlines the multiple imputation model. We chose to model marriage rates, rather than one-year change in marriage rates, because one-year change in marriage rates, one-year lag in marriage rates and the error correction term for marriage rates are all defined by marriage rates; after imputation, each of these variables were redefined in terms of the five imputed values of marriage rates. Quadratic polynomial effects of time were included, and permitted to vary across states.

(S1)

Ten imputed data sets were created, which was assumed sufficient, since the relative efficiency of basing our imputation on ten versus an infinite number of imputed data sets is approximately (1 + 0.0045/10)^–1^ = 0.9996 [S1]. Subsequently, the model described in the main text was fit to all ten imputed data sets; a combined estimate *θ*_c_ was the mean corresponding estimate across all ten, as in equation S2, and the standard error of a reported estimate *θ*_c_ was given by equation S3 [S2]. We employed a ridge prior [S3] equal to 1 (just under 2% of the number of time series), to aid in convergence given the colinearity of the policy change and lag variables.

(S2)

(S3)

Finally, the missing data are in California in 1991 a few years *before* California implemented same sex union laws, and in Oklahoma from 2000–2003, and Oklahoma never implemented same sex marriage or union laws. The uncertainty introduced into our model from data missingness is likely to produce only a miniscule effect in the estimate of the error correction process, and to produce very little change in the estimates of the effects of same sex marriage and union laws.

Table S2: Fixed and random effect model estimates of change in opposite sex marriage rates by state and year (*N*=1071).

|  | estimate^a^ | s.e.^b^ | 95% CI^c^ | | *q*-values^d^ |
| --- | --- | --- | --- | --- | --- |
| *Fixed effect estimates* |  |  |  |  |  |
| equilibrium correction rate | -0.0419 | 0.0029 | -0.0477 | -0.0362 | <0.0001 |
| change in same sex marriage law | 0.0001 | 0.0013 | -0.0025 | 0.0027 | >0.9999 |
| 1-year lag of same sex marriage law | -0.0421 | 0.0030 | -0.0480 | -0.0361 | <0.0001 |
| change in strong same sex union law | -0.0003 | 0.0007 | -0.0016 | 0.0010 | >0.9999 |
| 1-year lag of strong same sex union law | -0.0422 | 0.0030 | -0.0480 | -0.0364 | <0.0001 |
| change in weak same sex union law | -0.0004 | 0.0006 | -0.0016 | 0.0008 | >0.9999 |
| 1-year lag of weak same sex union law | -0.0421 | 0.0030 | -0.0479 | -0.0362 | <0.0001 |
| change in marriages * strong unions | -0.0005 | 0.0018 | -0.0041 | 0.0031 | >0.9999 |
| 1-year lag of marriages * strong unions | -0.0426 | 0.0044 | -0.0513 | -0.0340 | <0.0001 |
| model constant | 0.0003 | 0.0001 | 0.0002 | 0.0004 | <0.0001 |
|  |  |  |  |  |  |
| *Random effect estimates* |  |  |  |  |  |
| state level constant term | 4.42*10^-15^ | 1.50*10^-14^ | 5.78*10^-18^ | 3.38*10^-12^ | >0.9999 |
| period-level residual | 0.0013 | <0.0001 | 0.0013 | 0.0014 | <0.0001 |

^a^ The arithmetic mean of the estimates from all ten imputed data sets.

^b^ Combined standard errors account for both within- and between-imputation estimate variance.

^c^ 95% confidence intervals are given by the estimate ± 1.96*s.e..

^d^ i.e. *p*-values adjusted upward to account for twelve multiple comparisons; both *q*-values for fixed effects and *q*-values for random effects should be compared to *α* (i.e. *q*-values for fixed effects are adjustments to 2**p*).

*Figure S1* Projected differences in annual opposite sex marriages in states enacting same sex marriage or strong or weak same sex union laws.


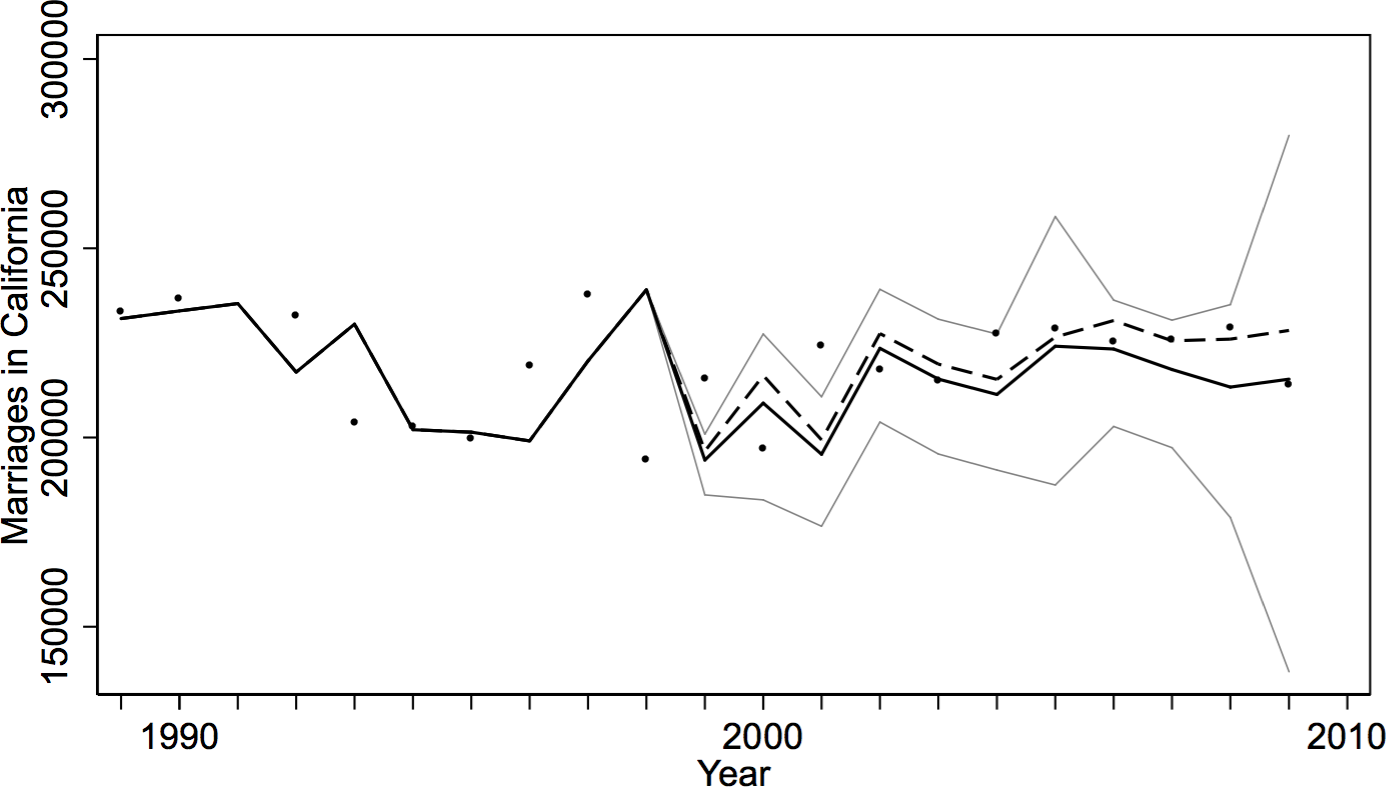

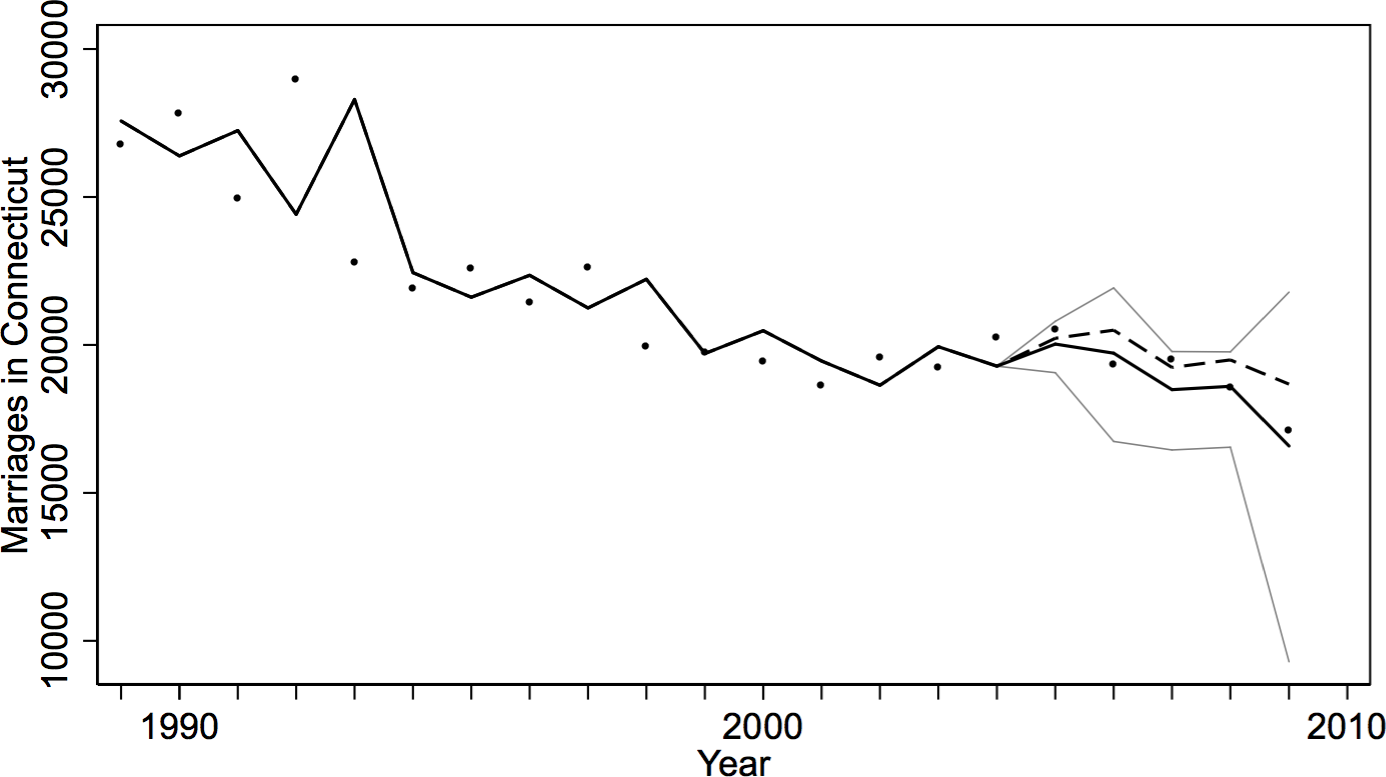

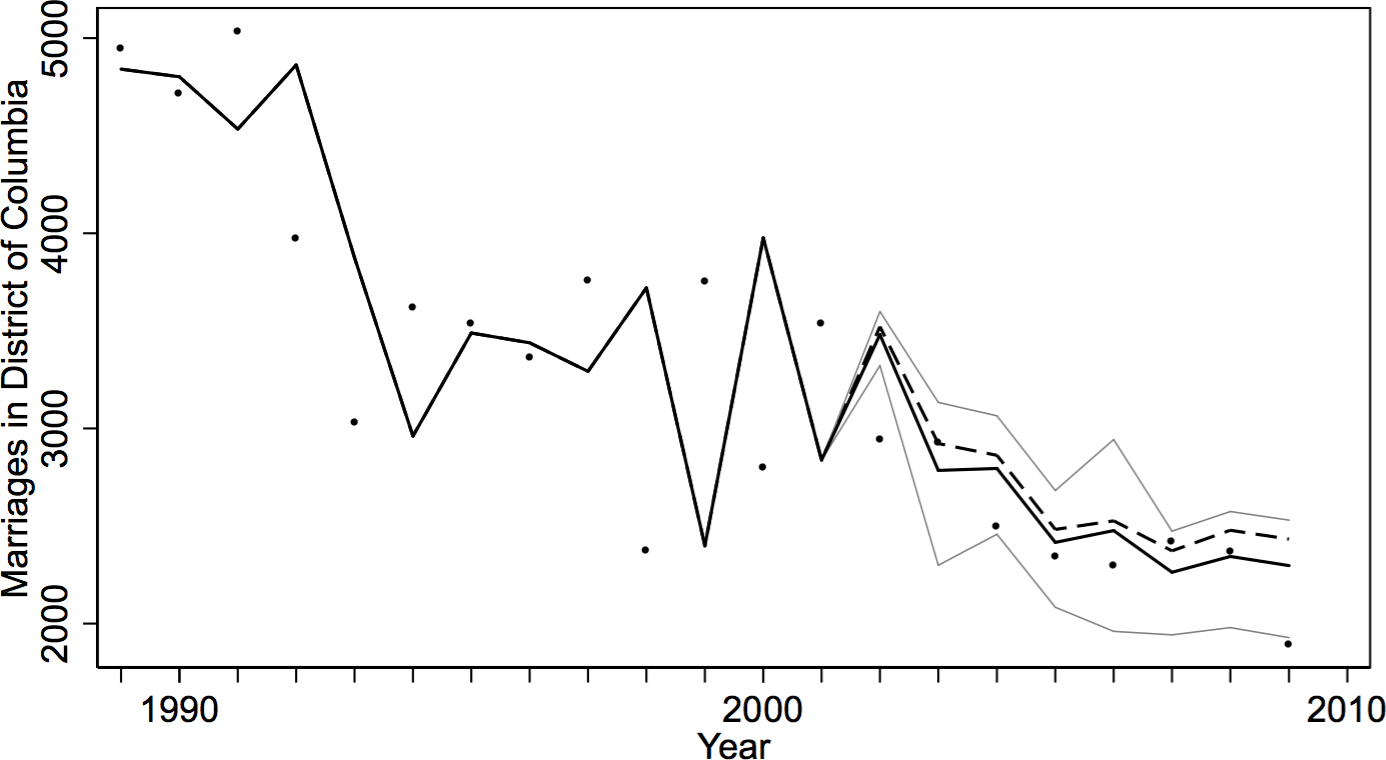


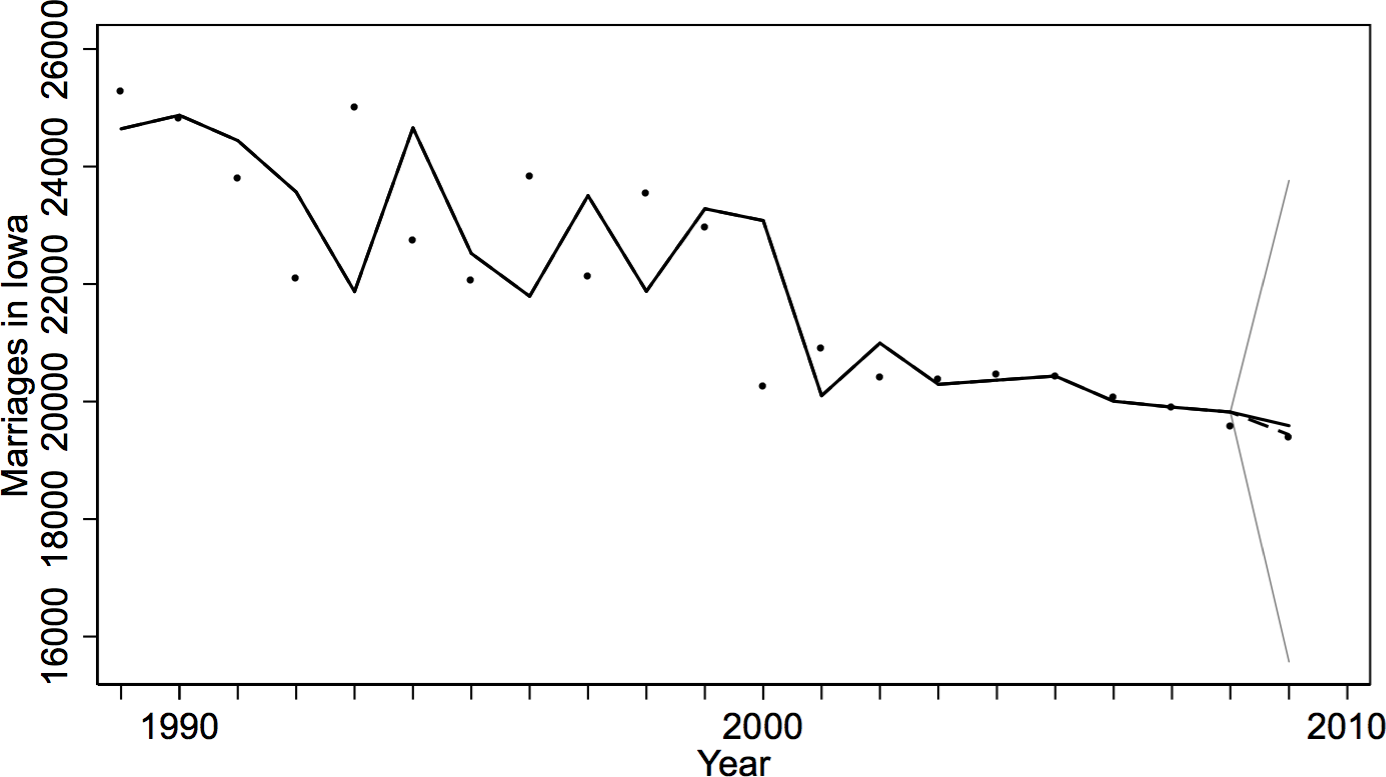

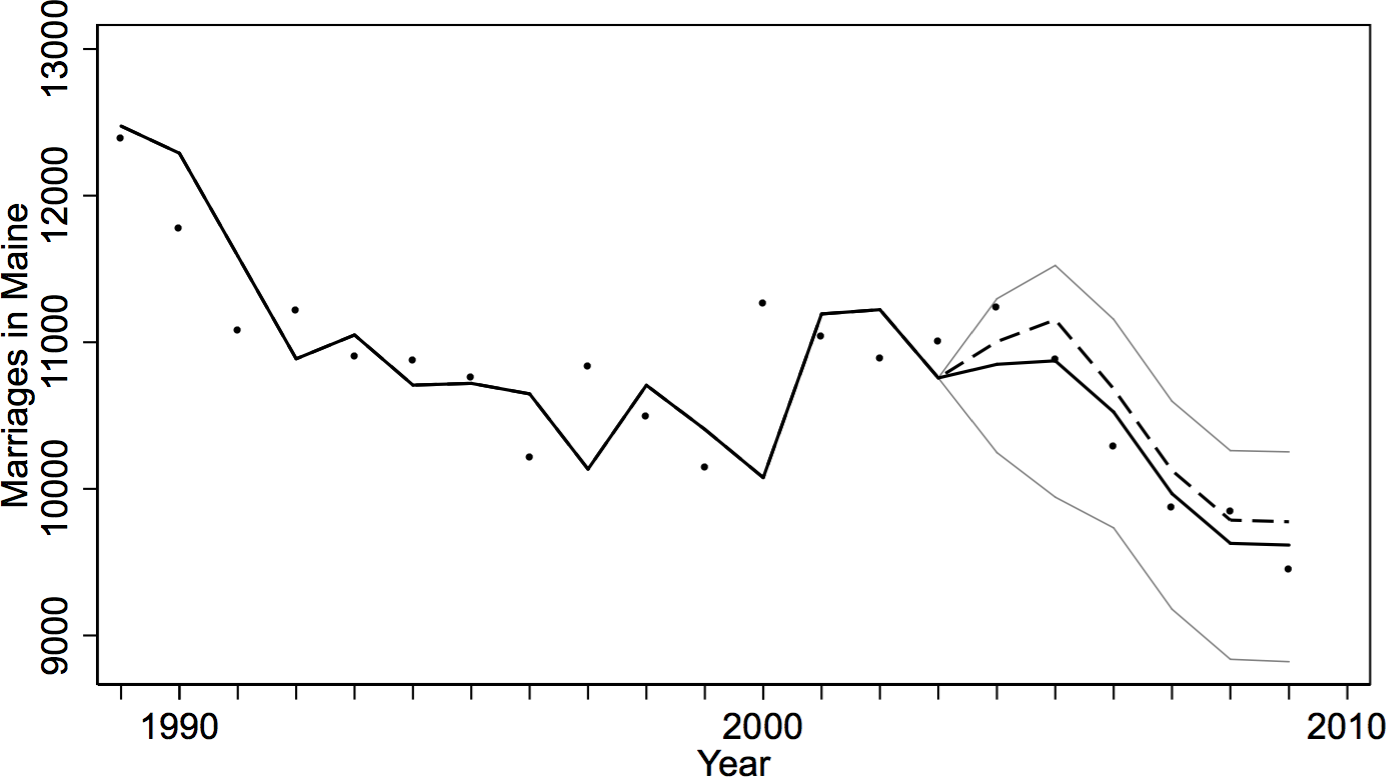

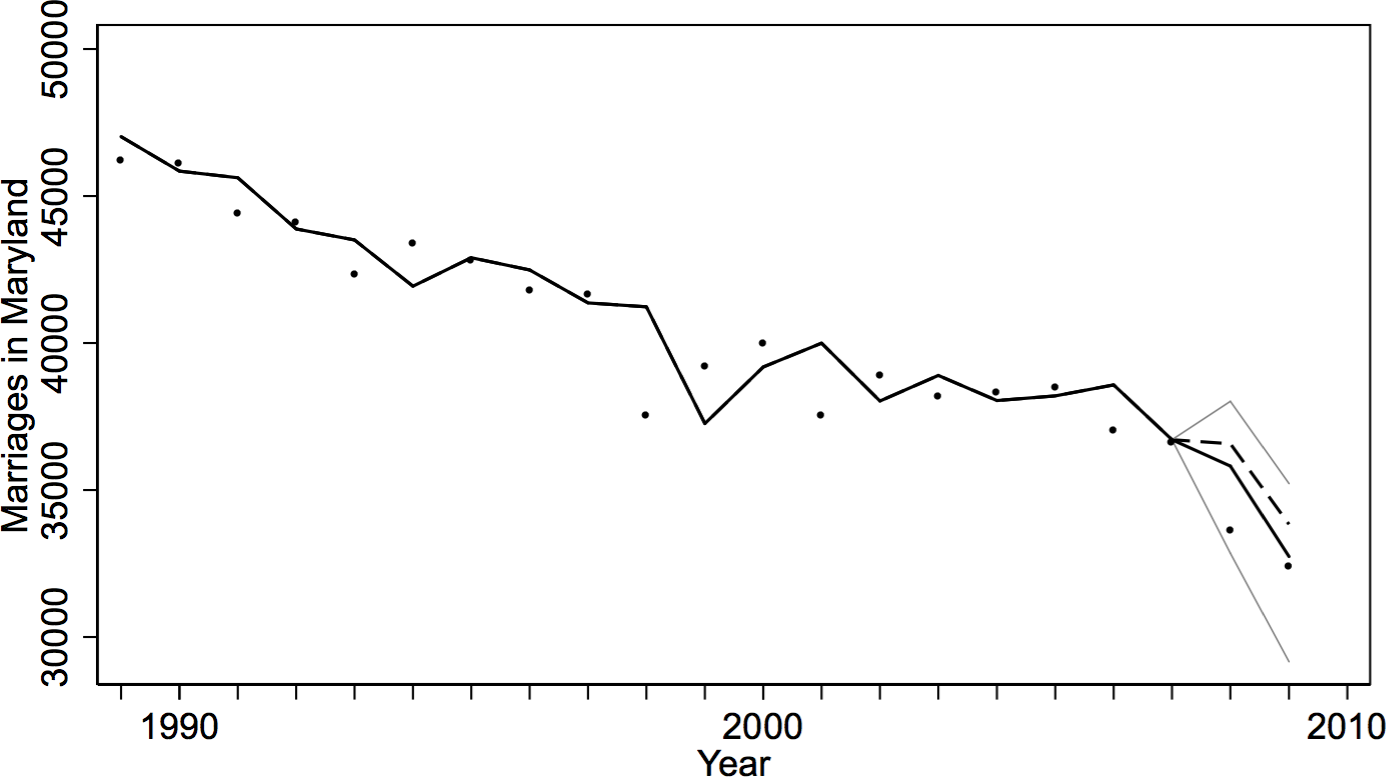


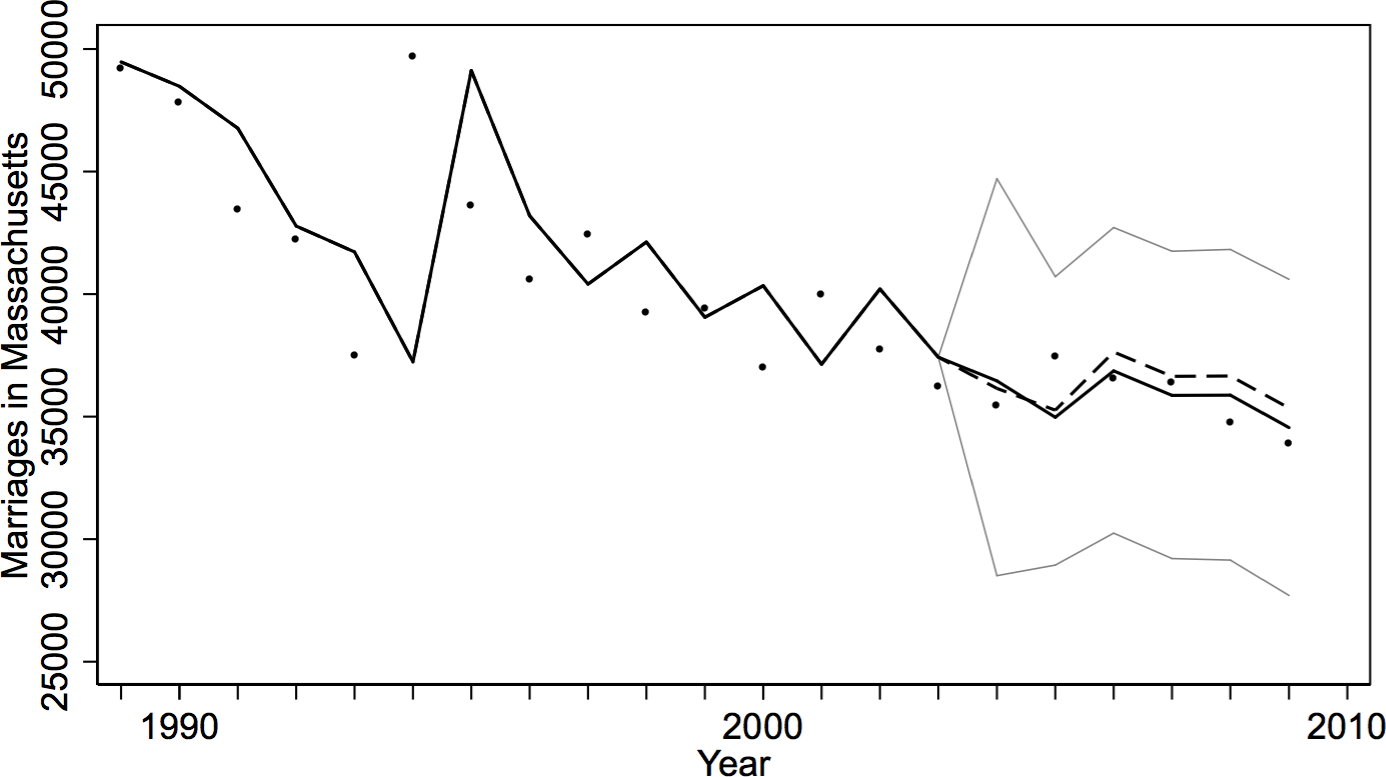

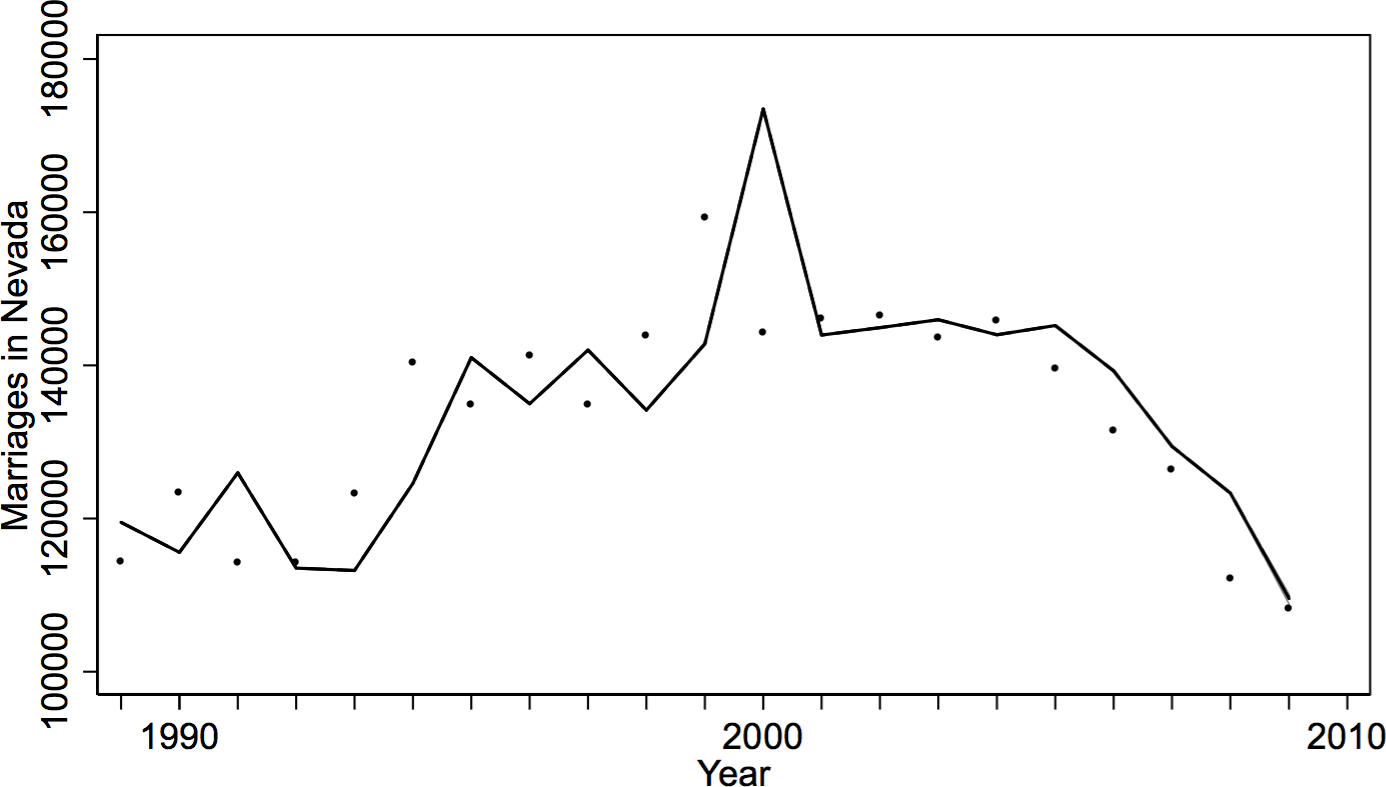

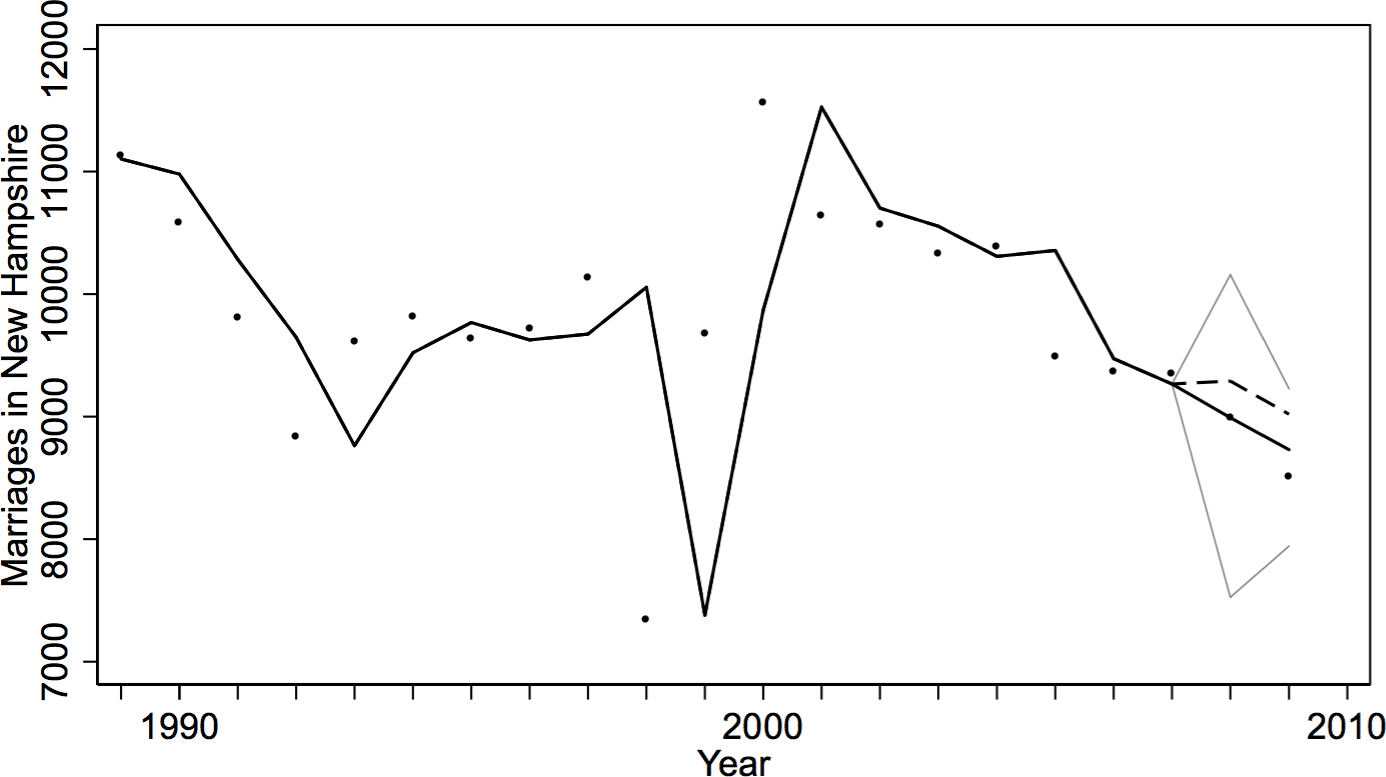


*Figure S1, continued*


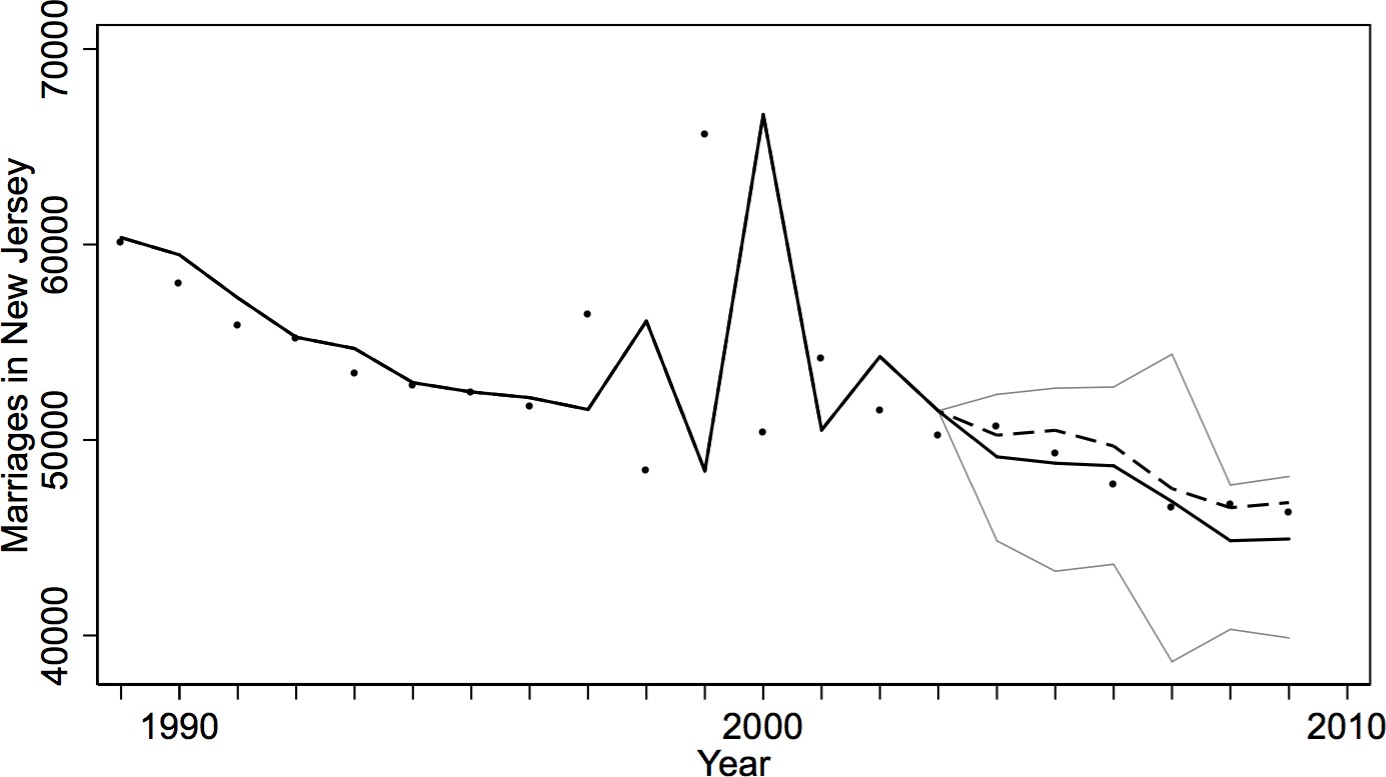

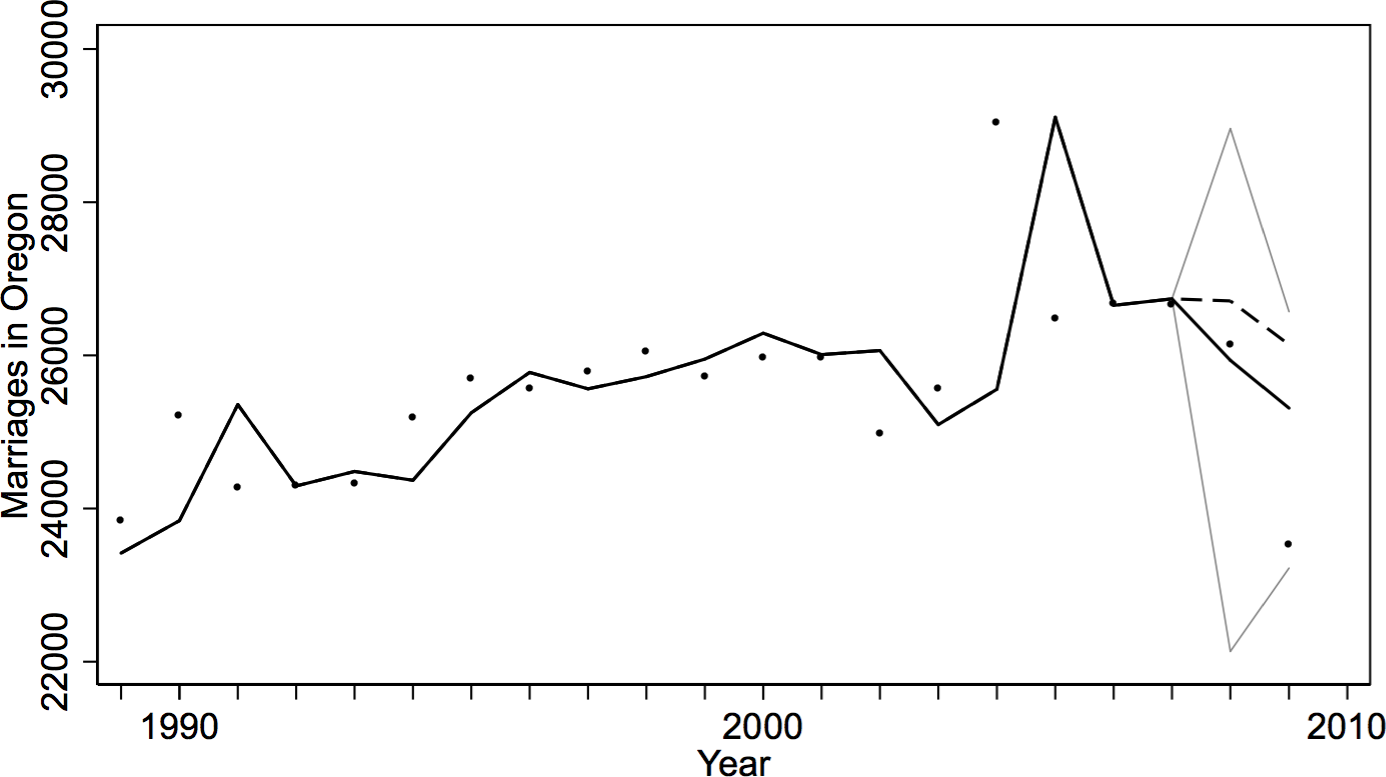

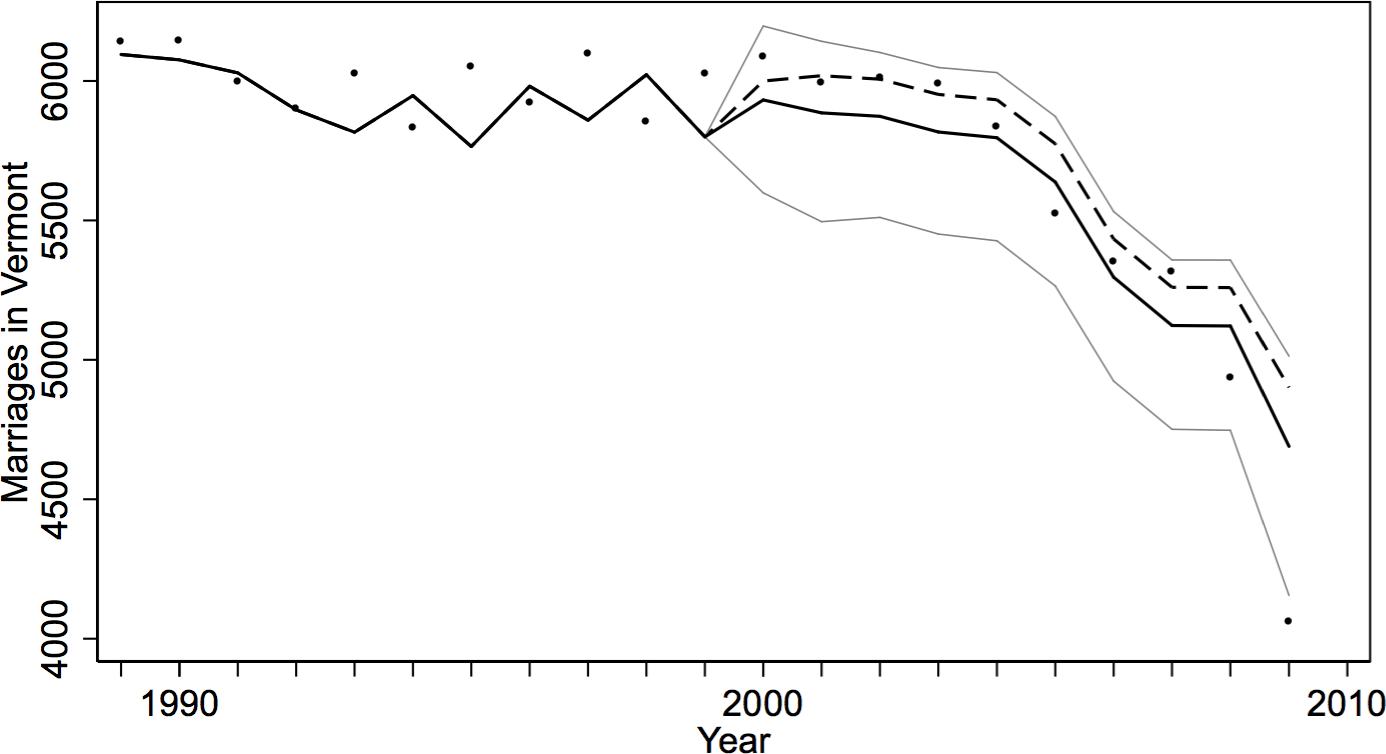


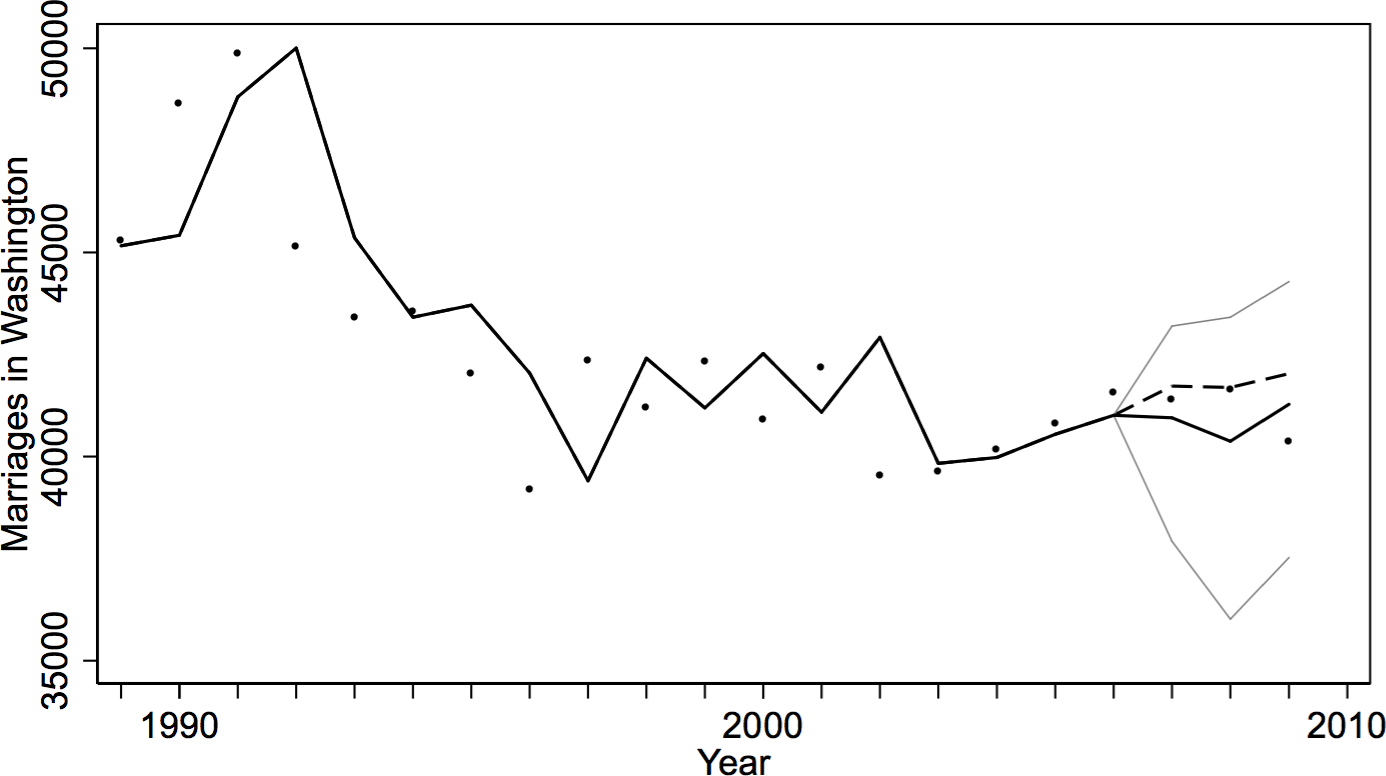

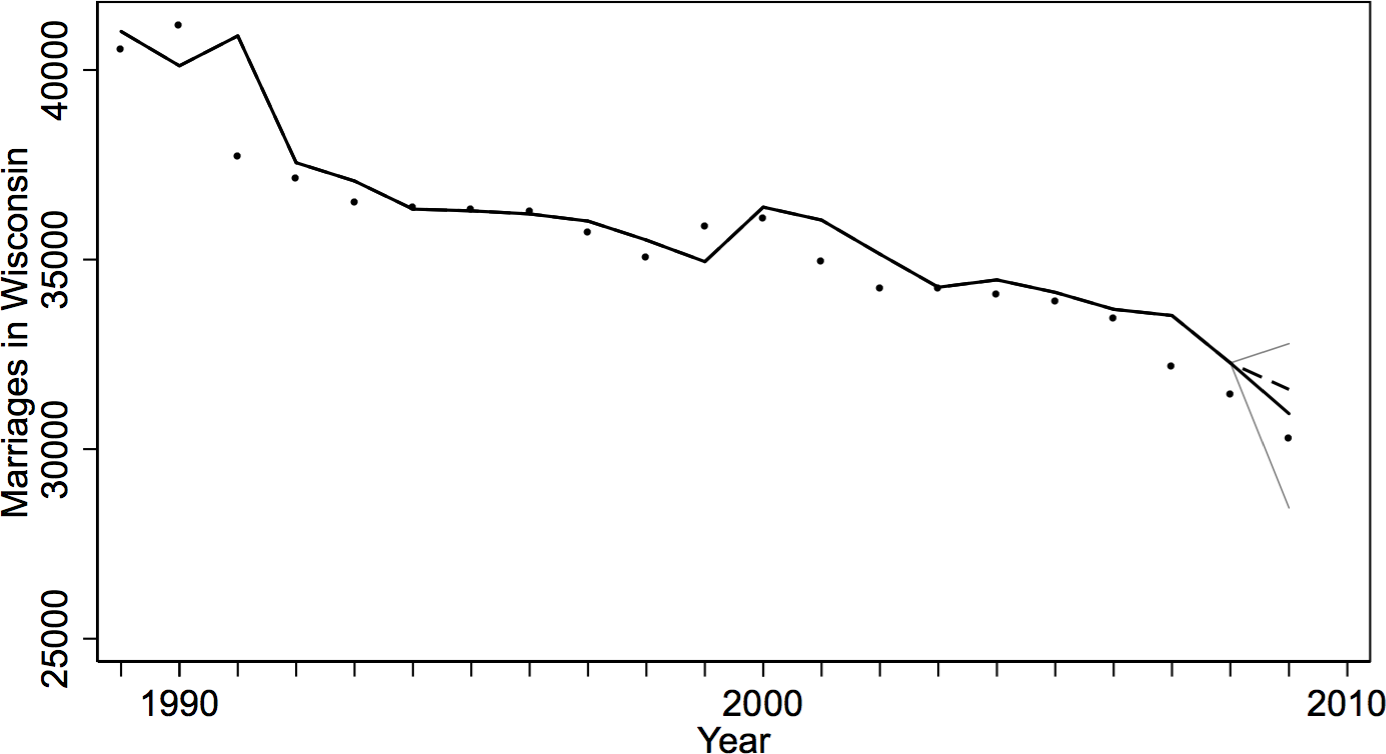


Figure S1 caption: Solid lines represent our modeled marriages in each year and state with the associated 95% prediction intervals. The dashed lines project opposite sex marriages if same sex marriage and strong or weak same sex union laws had not been enacted in each state and year.

*Separate generalized error correction models for same sex marriage and strong and weak same sex union laws*

Equation (2) from the main text assumes that the error correction rate is homogenous for same sex marriages and for strong and weak same sex unions. We examined this assumption by estimating the error correction parameter of equations (see Tables S3, S5 and S7). The results combined estimates of were -0.0419 (s.d. 0.0029), -0.0418 (s.d. 0.0029) and -0.0417 (s.d. 0.0029) for models S4, S5 and S6 respectively (see Tables S4, S6 and S8).

(S4)
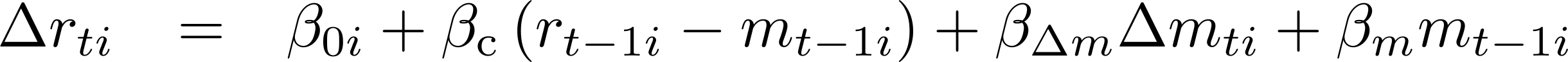


(S5)
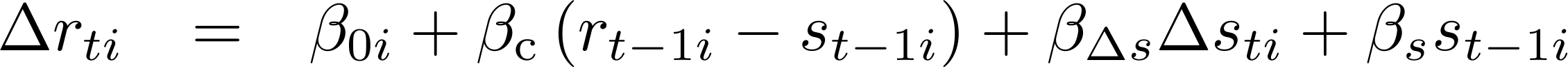


(S6)
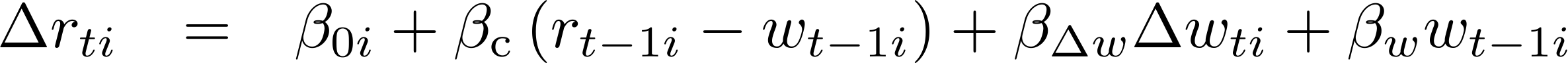


Table S3: Effects of only same sex marriage laws on opposite sex marriage rates (*N*=1071).

|  | estimate^a^ | s.e.^b^ | 95% CI^c^ | | *q*-values^d^ |
| --- | --- | --- | --- | --- | --- |
| *Effects of same sex marriage:* |  |  |  |  |  |
| Instantaneous short run effect | -0.0003 | 0.0009 | -0.0021 | 0.0014 | >0.9999 |
| Lagged short run effect | 0.0001 | 0.0011 | -0.0020 | 0.0023 | >0.9999 |
| Long run effect | -0.0043 | 0.0149 | -0.0336 | 0.0249 | >0.9999 |

^a^ The arithmetic mean of the estimates from all ten imputed data sets.

^b^ Combined standard errors account for both within- and between-imputation estimate variance.

^c^ 95% confidence intervals are given by the estimate ± 1.96*s.e..

^d^ i.e. *p*-values adjusted upward to account for three multiple comparisons that should be compared to *α* (i.e. *q*-values for fixed effects are adjustments to 2**p*).

Table S4: Fixed and random effect model estimates of change in opposite sex marriage rates by state and year for same sex marriage only (*N*=1071).

|  | estimate^a^ | s.e.^b^ | 95% CI^c^ | | *q*-values^d^ |
| --- | --- | --- | --- | --- | --- |
| *Fixed effect estimates* |  |  |  |  |  |
| equilibrium correction rate | -0.0417 | 0.0029 | -0.0474 | -0.0359 | <0.0001 |
| change in same sex marriage law | -0.0003 | 0.0009 | -0.0021 | 0.0014 | >0.9999 |
| 1-year lag of same sex marriage law | -0.0418 | 0.0030 | -0.0477 | -0.0359 | <0.0001 |
| model constant | 0.0003 | 0.0001 | 0.0002 | 0.0004 | <0.0001 |
|  |  |  |  |  |  |
| *Random effect estimates* |  |  |  |  |  |
| state level constant term | 5.20*10^-15^ | 1.69*10^-14^ | 8.72*10^-18^ | 3.10*10^-12^ | >0.9999 |
| period-level residual | 1.33*10^-03^ | 2.89*10^-05^ | 1.27*10^-03^ | 1.38*10^-03^ | <0.0001 |

^a^ The arithmetic mean of the estimates from all ten imputed data sets.

^b^ Combined standard errors account for both within- and between-imputation estimate variance.

^c^ 95% confidence intervals are given by the estimate ± 1.96*s.e..

^d^ i.e. *p*-values adjusted upward to account for six multiple comparisons; both *q*-values for fixed effects and *q*-values for random effects should be compared to *α* (i.e. *q*-values for fixed effects are adjustments to 2**p*).

Table S5: Effects of only strong same sex union laws on opposite sex marriage rates (*N*=1071).

|  | estimate^a^ | s.e.^b^ | 95% CI^c^ | | *q*-values^d^ |
| --- | --- | --- | --- | --- | --- |
| *Effects of strong same sex union:* |  |  |  |  |  |
| Instantaneous short run effect | -0.0002 | 0.0006 | -0.0013 | 0.0009 | >0.9999 |
| Lagged short run effect | -0.0001 | 0.0007 | -0.0014 | 0.0012 | >0.9999 |
| Long run effect | -0.0078 | 0.0069 | -0.0214 | 0.0058 | >0.9999 |

^a^ The arithmetic mean of the estimates from all ten imputed data sets.

^b^ Combined standard errors account for both within- and between-imputation estimate variance.

^c^ 95% confidence intervals are given by the estimate ± 1.96*s.e..

^d^ i.e. *p*-values adjusted upward to account for three multiple comparisons that should be compared to *α* (i.e. *q*-values for fixed effects are adjustments to 2**p*).

Table S6: Fixed and random effect model estimates of change in opposite sex marriage rates by state and year for strong same sex unions only (*N*=1071).

|  | estimate^a^ | s.e.^b^ | 95% CI^c^ | | *q*-values^d^ |
| --- | --- | --- | --- | --- | --- |
| *Fixed effect estimates* |  |  |  |  |  |
| equilibrium correction rate | -0.0418 | 0.0029 | -0.0475 | -0.0360 | <0.0001 |
| change in strong same sex union law | -0.0002 | 0.0006 | -0.0013 | 0.0009 | >0.9999 |
| 1-year lag of strong same sex union law | -0.0421 | 0.0030 | -0.0479 | -0.0363 | <0.0001 |
| model constant | 0.0003 | 0.0001 | 0.0002 | 0.0004 | <0.0001 |
|  |  |  |  |  |  |
| *Random effect estimates* |  |  |  |  |  |
| state level constant term | 4.65*10^-15^ | 1.50*10^-14^ | 8.23*10^-18^ | 2.63*10^-12^ | >0.9999 |
| period-level residual | 1.32*10^-03^ | 2.89*10^-05^ | 1.27*10^-03^ | 1.38*10^-03^ | <0.0001 |

^a^ The arithmetic mean of the estimates from all ten imputed data sets.

^b^ Combined standard errors account for both within- and between-imputation estimate variance.

^c^ 95% confidence intervals are given by the estimate ± 1.96*s.e..

^d^ i.e. *p*-values adjusted upward to account for six multiple comparisons; both *q*-values for fixed effects and *q*-values for random effects should be compared to *α* (i.e. *q*-values for fixed effects are adjustments to 2**p*).

Table S7: Effects of only weak same sex union laws on opposite sex marriage rates (*N*=1071).

|  | estimate^a^ | s.e.^b^ | 95% CI^c^ | | *q*-values^d^ |
| --- | --- | --- | --- | --- | --- |
| *Effects of weak same sex union law:* |  |  |  |  |  |
| Instantaneous short run effect | -0.0002 | 0.0006 | -0.0013 | 0.0009 | >0.9999 |
| Lagged short run effect | 0.0000 | 0.0006 | -0.0012 | 0.0012 | >0.9999 |
| Long run effect | -0.0032 | 0.0084 | -0.0196 | 0.0131 | >0.9999 |

^a^ The arithmetic mean of the estimates from all ten imputed data sets.

^b^ Combined standard errors account for both within- and between-imputation estimate variance.

^c^ 95% confidence intervals are given by the estimate ± 1.96*s.e..

^d^ i.e. *p*-values adjusted upward to account for three multiple comparisons that should be compared to *α* (i.e. *q*-values for fixed effects are adjustments to 2**p*).

Table S8: Fixed and random effect model estimates of change in opposite sex marriage rates by state and year for weak same sex unions only (*N*=1071).

|  | estimate^a^ | s.e.^b^ | 95% CI^c^ | | *q*-values^d^ |
| --- | --- | --- | --- | --- | --- |
| *Fixed effect estimates* |  |  |  |  |  |
| equilibrium correction rate | -0.0417 | 0.0029 | -0.0474 | -0.0360 | <0.0001 |
| change in same sex weak union law | -0.0002 | 0.0006 | -0.0013 | 0.0009 | >0.9999 |
| 1-year lag of same sex weak union law | -0.0419 | 0.0030 | -0.0477 | -0.0361 | <0.0001 |
| model constant | 0.0003 | 0.0001 | 0.0002 | 0.0004 | <0.0001 |
|  |  |  |  |  |  |
| *Random effect estimates* |  |  |  |  |  |
| state level constant term | 5.36*10^-15^ | 1.71*10^-14^ | 1.02*10^-17^ | 2.81*10^-12^ | >0.9999 |
| period-level residual | 1.33*10^-03^ | 2.89*10^-05^ | 1.27*10^-03^ | 1.38*10^-03^ | <0.0001 |

^a^ The arithmetic mean of the estimates from all ten imputed data sets.

^b^ Combined standard errors account for both within- and between-imputation estimate variance.

^c^ 95% confidence intervals are given by the estimate ± 1.96*s.e..

^d^ i.e. *p*-values adjusted upward to account for six multiple comparisons; both *q*-values for fixed effects and *q*-values for random effects should be compared to *α* (i.e. *q*-values for fixed effects are adjustments to 2**p*).

**References S1**

[S1] Rubin, D. B. (1987). *Imputation for Nonresponse in Surveys*. John, Wiley and Sons, Inc.

[S2] Schafer, J. L. (1999). Multiple imputation: a primer. *Statistical Methods in Medical Research*, 8:3–15.

[S3] Honaker, J. and King, G. (2010). What to do about missing values in time-series cross-section data. *American Journal of Political Science*, 54(2):561–581.
